# Clinical study of ultrasonic evaluation of T/N staging of differentiated thyroid carcinoma using AJCC 8^th^ staging criteria

**DOI:** 10.1371/journal.pone.0269994

**Published:** 2022-06-16

**Authors:** Yu Liang, Xingxiang Huang, Zhe Song, Yang Yang, Ju Lei, Mei Ren, Li Tan, Hui Zhang

**Affiliations:** 1 Department of Ultrasound, Sichuan Academy of Medical Sciences & Sichuan Provincial People’s Hospital, School of Medicine, University of Electronic Science and Technology of China, Chengdu, Sichuan, China; 2 School of Medicine, University of Electronic Science and Technology of China, Chengdu, Sichuan, China; 3 Department of Thyroid Surgery, Sichuan Academy of Medical Sciences & Sichuan Provincial People’s Hospital, School of Medicine, University of Electronic Science and Technology of China, Chengdu, Sichuan, China; 4 North Sichuan Medical College, Nanchong, Sichuan, China; Brigham and Women’s Hospital, Harvard Medical School, UNITED STATES

## Abstract

**Objective:**

To explore the value of ultrasound in evaluating T/N staging of differentiated thyroid carcinoma (DTC).

**Methods:**

The clinical data of 1206 patients with DTC in our hospital from January 2018 to December 2020 were retrospectively analyzed. Cervical ultrasound was performed before surgery, and the standard ultrasound images of thyroid nodules and cervical lymph nodes I to VII were retained. According to the 8th TNM staging guidelines of AJCC DTC, the T/N stages were assessed by preoperative ultrasonic data. Then, the sensitivity, specificity, negative predicted value, positive predicted value (PPV), and diagnostic value of ultrasound T/N staging were assessed using postoperative pathological staging as the reference.

**Results:**

Ultrasonic T-stage had good consistency to pathological T stage in T4a and T4b tumors (kappa value>0.75), and moderate consistency to pathological T stage in T1, T2 and T3a tumors (kappa value between 0.4 and 0.75). ultrasonic T-stage had a sensitivity higher than 66%, except in T3b assessment (13/44, 29.5%, 95%CI: 16.1%-43.0%). All ultrasonic T-stage had specificity higher than 93%, except in T1b assessment (734/889, 82.6%, 95%CI: 80.1%-85.1%). The PPV of ultrasonic T1a to T4b was 94.3% (494/524), 61.0% (242/397), 54.4% (87/160), 34.3% (12/35), 20.3% (13/64), 100% (22/22) and 100% (4/4), respectively. The diagnostic accuracy values were 83% in T1a, 81% in T1b, 91% in T2, 98% in T3a, 93% in T3b, 99% in T4a and 100% in T4b. Nltrasonic N-stage had poor consistency to pathological N stage in any N stages (kappa value<0.3). The PPV of ultrasonic N0, N1, N1a and N1b was 61.0% (542/889), 55.2% (37/67), 48.2% (53/110), and 24.3% (34/140), respectively.

**Conclusion:**

Ultrasound has a good consistency and high accuracy in assessing the T-stage of DTC. However, the consistency and accuracy were poor in N-staging. It has a certain reference value in reducing excessive surgical treatment of DTC.

## Introduction

Thyroid carcinoma (TC) has become the fifth-highest incidence rate of cancer globally [1]. Among them, more than 95% are differentiated thyroid carcinoma (DTC) [2]. DTC is prone to develop regional lymph node metastasis, but the degree of malignancy is very low. There is a trend of overtreatment of this disease in the world [3]. Accurate evaluation of DTC staging is of great clinical significance for selecting treatment schemes [4]. At present, surgeons mainly judge the malignant risk of thyroid nodules and decide the treatment strategy by TI-RADS classification reported by thyroid ultrasound. However, TI-RADS classification can only evaluate the imaging risk of benign and malignant thyroid nodules. It cannot evaluate cervical lymph nodes around the thyroid and distant metastasis, which is a great limitation in the clinical evaluation of DTC [4–6]. In 2017, the American Joint Committee on Cancer (AJCC) issued the latest staging system for DTC TNM-8 [7], which comprehensively evaluates the malignant risk of DTC and is more conducive to accurate diagnosis and treatment. M-stage distant metastasis is most common in lung, bone and other organs that are not suitable for ultrasonography. Therefore, this study focused on whether cervical ultrasonography can help clinicians evaluate the T/N staging of DTC.

## Materials and methods

### Data retrieving

A retrospective analysis was conducted based on patients’ records in Sichuan Academy of Medical Sciences & Sichuan Provincial People’s Hospital, School of Medicine, Chengdu, Sichuan, China. All data were fully anonymized and the ethics committee of our hospital waived the requirement for informed consent. The following inclusion criteria were applied to identify DTC cases for this study: (1) DTC patients confirmed by our hospital and confirmed via postoperative pathology; (2) All patients underwent preoperative thyroid and cervical lymph node ultrasonography in our hospital within two months after ultrasonic check, with clear and complete ultrasonic check data; (3) The clinical history, physical examination and laboratory examination (thyroid hormone, CEA, calcitonin, etc.) of DTC patients are completed. Any cases meeting the following exclusion criteria were excluded: (1) thyroid nodule biopsy cytology showed that DTC was not operated; (2) The postoperative pathology was myeloid carcinoma, undifferentiated carcinoma, mesenchymal tissue-derived sarcoma, lymphoma and metastatic malignant tumor. This retrospective study was approved by the ethics committee of Sichuan Provincial People’s Hospital, China.

### TNM staging criteria for thyroid carcinoma

TNM staging was performed in patients with DTC based on the 8th edition of the AJCC staging system for thyroid cancer (AJCC TNM-8) [8].

### Ultrasound imaging criteria for T/N staging of DTC

Ultrasound check was conducted using ultrasonographic scanners (Accuvix A30/ Samsung Medison, Aixplorer/SuperSonic Imagine and EPIQ5/Philips) equipped with a linear transducer (10–12 MHz) for morphological studies and a 4.7 MHz transducer for color-Doppler evaluation. All patients received scanning in supine position with hyperextended neck, in transverse and longitudinal planes. Real time imaging of thyroid lesions is visualized using both gray-scale and color Doppler techniques. The imaging characteristics of a mass, including shape, locations, size, margin (well defined or blurred), vascular pattern, contents (presence/absence of calcification) and echogenicity (isoechoic, hyper-or hypo-echoic) were checked and recorded.

Referring to the AJCC TNM-8 staging standard [8], we established the following ultrasonic T/N staging criteria (Tables 1 and 2). Thyroid ultrasound T-stage is based on the size, location of thyroid nodules and their relationship with surrounding tissues and organs.

**Table 1:** Ultrasonic T staging criteria.

T stages	Staging criteria
**TX**	No evidence of primary tumor.
**T1**	Tumor ≤2 cm in greatest dimension limited to the thyroid.
T1a	Tumor ≤1 cm in greatest dimension limited to the thyroid.
T1b	Tumor>1 cm but ≤2 cm in greatest dimension limited to the thyroid.
**T2**	Tumor>2 cm but ≤4 cm in greatest dimension limited to the thyroid.
**T3**	Tumor>4 cm limited to the thyroid, or gross extrathyroidal extension invading only strap muscles.
T3a	Tumor>4 cm limited to the thyroid.
T3b	Gross extrathyroidal extension invading only strap muscles (sternohyoid, sternothyroid, or omohyoid muscles) from a tumor of any size.
**T4**	Includes gross extrathyroidal extension beyond the strap muscles.
T4a	Gross extrathyroidal extension invading subcutaneous soft tissues, larynx, trachea, esophagus, or recurrent laryngeal nerve from a tumor of any size.
T4b	Thyroid nodules of any size invaded and grew into the prevertebral fascia or wrapped around the carotid artery or mediastinal vessels.

The N-stage of neck ultrasound is based on the signs of metastasis in the ultrasonic image characteristics of neck lymph nodes: microcalcification, cystic change, hyperecho and peripheral blood flow in lymph nodes. In addition, it also includes the round shape of lymph nodes, irregular or fuzzy boundary, uneven internal echo, disappearance of lymph hilus or unclear boundary between skin and medulla, etc.

**Table 2:** Ultrasonic N staging standard.

N stages	Staging criteria
**NX**	Regional lymph nodes cannot be assessed
**N0**	No evidence of locoregional lymph node metastasis
N0a	One or more cytologically or histologically confirmed benign lymph nodes
N0b	No radiologic or clinical evidence of locoregional lymph node metastasis
**N1**	Metastasis to regional nodes
N1a	Metastasis to level VI or VII (pretracheal, paratracheal, or prelaryngeal/Delphian, or upper mediastinal) lymph nodes. This can be unilateral or bilateral disease.
N1b	Metastasis to unilateral, bilateral, or contralateral later neck lymph nodes (levels I, II, III, IV, or V) or retropharyngeal lymph nodes

One ultrasound doctor and one surgeon in our hospital performed T/N staging of ultrasound and pathological results of DTC patients by a double-blind method. The inconsistency was solved by discussion.

### Statistical analysis

The data obtained in this study were statistically analyzed by SPSS 19.0. The counting data were expressed in examples (percentage) and the measurement data were expressed in mean ± standard deviation (SD). Diagnostic test 2 × 2 tables were generated for the assessment of sensitivity, specificity, negative predicted value, positive predicted value, and diagnostic value ultrasound T/N stages compared to the postoperative pathological TNM stages (used as the reference). The following formula were applied: sensitivity = [TP/(TP + FN)] × 100%; specificity = [TN/(FP + TN)] × 100%; positive predicted value (PPV) = [TP/(TP + FP)] × 100%; negative predicted value (NPV) = [TN/(TN + FN)] × 100%; and diagnostic accuracy (DA) = [(TP + TN)/(TP + TN + FP + FN)] × 100%, in which TP refers true positive, TN refers true negative, FP refers false positive, and FN refers false negative. The consistency between the clinical T/N stage and pathological T/N stage was analyzed by calculating the kappa value. P<0.05 was considered statistically different.

## Results

### Patient clinical parameters

From January 2018 to December 2020, a total of 1823 patients underwent surgical treatment in Sichuan Provincial People’s hospital due to suspected malignant thyroid nodules, of which 1235 patients were confirmed as malignant thyroid tumors by postoperative pathology. 1206 patients met the conditions and were included in this study, including 338 males and 868 females. The clinical parameters of DTC patients included in this study were summarized in Table 3 below:

**Table 3:** Clinical parameters of DTC patients.

Clinical parameters	Number of cases (%)
**Gender:** male/female	338 (28.0)/868 (72.0)
**Age (year)**	41.9 ± 12.1
Ultrasound showed nodule size (mm, mean ± SD)	14.5 ± 10.2
Ultrasonography showed single focus lesions	618 (51.2)
Ultrasound showed extravasation of capsule	163 (13.5)
Ultrasonography showed cervical lymph node metastasis	320 (26.5)
**Ultrasonic TI-RADS classification**	
Category 3	86 (7.1)
Category 4A	76 (6.3)
Category 4B	321 (26.6)
Category 4C	393 (32.5)
Category 5	330 (27.3)
**Pathological subtype**	
Papillary carcinoma	1179 (97.8)
follicular carcinoma	27 (2.2)
**Pathology showed extravasation of capsule**	81 (6.7)
**Pathology showed cervical lymph node metastasis**	588 (48.7)

The details of all clinical parameters were provided in S1 Table. 727 cases (60.3%) were ≤ 45 years old, 479 cases (39.7%) were > 45 years old. The average age was (41.9 ± 12.1) years old. Thyroid ultrasound TI-RADS classification ≥ 4B in 1044 cases (86.6%), ultrasound showed extraglandular invasion in 163 cases (13.5%), and ultrasound showed cervical lymph node metastasis in 320 cases (26.5%). Postoperative pathology confirmed 1179 cases of papillary carcinoma (97.8%), 81 cases of extraglandular invasion (6.7%) and 588 cases of cervical lymph node metastasis (48.7%). All these patients received surgery within two months after ultrasonic check.

### Comparison of T-stage results

The consistency between ultrasonic T-stage and pathological T-stage is summarized in Table 4.

**Table 4:** The consistency between ultrasonic T-stages and pathological T-stages of DTC.

Ultrasonic T-stage	No. of cases	Pathological T-stage							Kappa value
		1a	1b	2	3a	3b	4a	4b	
1a	524 (43%)	494 (41%)	22 (2%)	3 (0%)	0 (0%)	5 (0%)	0 (0%)	0 (0%)	0.66
1b	397 (33%)	127 (11%)	242 (20%)	16 (1%)	1 (0%)	11 (1%)	0 (0%)	0 (0%)	0.54
2	160 (13%)	19 (2%)	39 (3%)	87 (7%)	0 (0%)	11 (1%)	4 (0%)	0 (0%)	0.57
3a	35 (3%)	10 (1%)	1 (0%)	8 (1%)	12 (1%)	4 (0%)	0 (0%)	0 (0%)	0.49
3b	64 (5%)	24 (2%)	13 (1%)	7 (1%)	0 (0%)	13 (1%)	7 (1%)	0 (0%)	0.21
4a	22 (2%)	0 (0%)	0 (0%)	0 (0%)	0 (0%)	0 (0%)	22 (2%)	0 (0%)	0.80
4b	4 (0%)	0 (0%)	0 (0%)	0 (0%)	0 (0%)	0 (0%)	0 (0%)	4 (0%)	1.00

**Fig 1:**
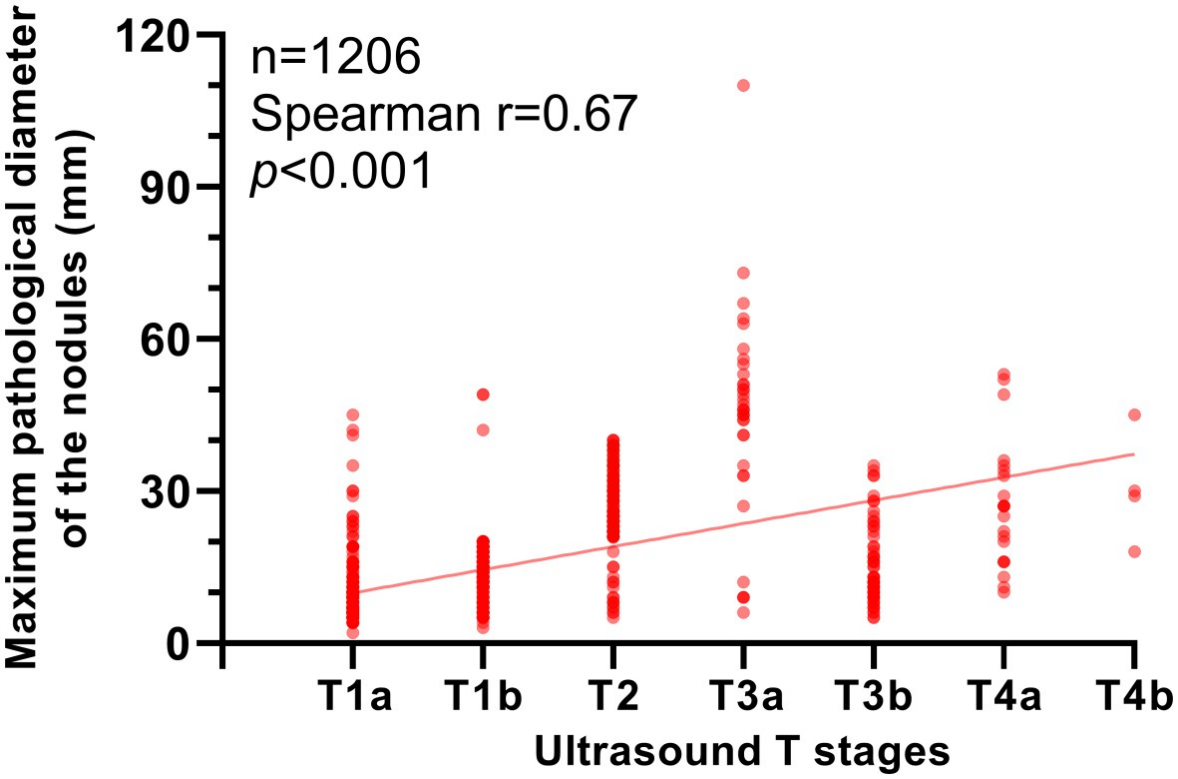
The correlation between maximum pathological diameter of the nodules (mm) and ultrasonic T-stages.

Pathological T stage confirmed that 92.2% (1112/1206) of the cases included in this study were in T1 and T2 stages. According to the calculation presented above, the ultrasonic T-stage had good consistency with the pathological T-stage in T4a and T4b tumors (kappa value>0.75). It had moderate consistency to pathological T stage in T1, T2 and T3a tumors (kappa value between 0.4 and 0.75). However, the consistency is poor in T3b tumors (kappa value = 0.21). The correlation between the maximum pathological diameter of the nodules (mm) and ultrasonic T-stage was assessed by Spearman’s correction. Results confirmed a strong positive correction (Spearman’s r = 0.67, *p*<0.001, Fig 1).

According the calculation presented above, ultrasonic T-stage had a sensitivity higher than 66%, except in T3b assessment (13/44, 29.5%, 95%CI: 16.1%-43.0%) (Table 5). All ultrasonic T-stage had specificity higher than 93%, except in T1b assessment (734/889, 82.6%, 95%CI: 80.1%-85.1%) (Table 5). The PPV of ultrasonic T1a to T4b was 94.3% (494/524), 61.0% (242/397), 54.4% (87/160), 34.3% (12/35), 20.3% (13/64), 100% (22/22) and 100% (4/4), respectively (Table 5). The diagnostic accuracy values were 83% in T1a, 81% in T1b, 91% in T2, 98% in T3a, 93% in T3b, 99% in T4a and 100% in T4b (Table 5).

**Table 5:** The diagnostic value of ultrasonic T-staging compared to pathological T-staging of DTC.

Ultrasonic T-stage	No. Sensitivity (95%CI)	No. Specificity (95%CI)	No. PPV (95%CI)	No. NPV (95%CI)	No. DA (%)
**1a**	494/674	502/532	494/524	502/682	996/1206
	73.3% (70.0%-76.6%)	94.4% (92.4%-96.3%)	94.3% (92.3%-96.3%)	73.6% (70.3%-76.9%)	83%
**1b**	242/317	734/889	242/397	734/809	976/1206
	76.3% (71.7%-81.0%)	82.6% (80.1%-85.1%)	61.0% (56.2%-65.8%)	90.7% (88.7%-92.7%)	81%
**2**	87/121	1012/1085	87/160	1012/1046	1099/1206
	71.9% (63.9%-80.0%)	93.3% (91.8%-94.8%)	54.4% (46.7%-62.1%)	96.7% (95.6%-97.8%)	91%
**3a**	12/13	1170/1193	12/35	1170/1171	1182/1206
	92.3% (77.8%-106.8%)	98.1% (97.3%-98.9%)	34.3% (18.6%-50.0%)	99.9% (99.7%-100.0%)	98%
**3b**	13/44	1111/1162	13/64	1111/1142	1124/1206
	29.5% (16.1%-43.0%)	95.6% (94.4%-96.8%)	20.3% (10.5%-30.2%)	97.3% (96.3%-98.2%)	93%
**4a**	22/33	1173/1173	22/22	1173/1184	1195/1206
	66.7% (50.6%-82.8%)	100% (100.0%-100.0%)	100% (100.0%-100.0%)	99.1% (98.5%-99.6%)	99%
**4b**	4/4	1202/1202	4/4	1202/1202	1206/1206
	100% (100.0%-100.0%)	100% (100.0%-100.0%)	100% (100.0%-100.0%)	100% (100.0%-100.0%)	100%

### Comparison of N-stage results

The consistency between ultrasonic N-stage and pathological N-stage are summarized in Table 6.

**Table 6:** The consistency between ultrasonic N-stages and pathological N-stages of DTC.

Ultrasonic N-stage	Number of cases	Pathological N-stage				Kappa value
		N0	N1a	N1b	N1	
N0	889	542	248	23	76	0.29
N1a	110	36	53	2	19	0.24
N1b	140	34	28	34	44	0.11
N1	67	6	12	12	37	0.26

According to the calculation presented above, the ultrasonic N-stage had poor consistency with the pathological N stage in all N stages (kappa value<0.3 in all N stages) (Table 6).

According to the calculations in Table 7, ultrasonic N-stage had sensitivity lower than 50% except in N1, N1a and N1b assessment (Table 7). Although its sensitivity was 87.7% (95%CI: 85.1%-90.3%) in N0, the specificity was only 41.0% (95%CI: 37.0%-45.0%) (Table 6). The PPV of ultrasonic N0, N1, N1a and N1b was 61.0% (542/889), 55.2% (37/67), 48.2% (53/110), and 24.3% (34/140), respectively (Table 7). The diagnostic accuracy values were 65% in N0, 86% in N1, 91% in T2, 71% in N1a and 88% N1b (Table 7). Thyroid ultrasound might help distinguish typical cervical lymph node metastasis for clinical diagnosis and treatment, but it should be interpreted cautiously.

**Table 7:** The diagnostic value of ultrasonic N-staging compared to pathological N-staging of DTC.

Ultrasonic T-stage	No. Sensitivity (95%CI)	No. Specificity (95%CI)	No. PPV (95%CI)	No. NPV (95%CI)	No. DA (%)
**N0**	542/618	241/588	542/889	241/317	783/1206
	87.7% (85.1%-90.3%)	41.0% (37.0%-45.0%)	61.0% (57.8%-64.2%)	76.0% (71.3%-80.7%)	65%
**N1**	37/176	1000/1030	37/67	1000/1139	1037/1206
	21.0% (15.0%-27.0%)	97.1% (96.1%-98.1%)	55.2% (43.3%-67.1%)	87.8% (85.9%-89.7%)	86%
**N1a**	53/341	808/865	53/110	808/1096	861/1206
	15.5% (11.7%-19.4%)	93.4% (91.2%-95.1%)	48.2% (38.8%-57.6%)	73.4% (71.1%-76.3%)	71%
**N1b**	34/71	1029/1135	34/140	1029/1066	1063/1206
	47.9% (36.3%-59.5%)	90.7% (89.0%-92.4%)	24.3% (17.2%-31.4%)	96.5% (95.4%-97.6%)	88%

## Discussion

In most hospitals in China, the preoperative evaluation of DTC mainly depends on thyroid ultrasound TI-RADS classification. The higher the classification, the greater risk of malignancy, and the greater probability of surgical treatment [9]. TI-RADS classification standard is only suitable for malignant risk assessment of specific ultrasonic signs of thyroid nodules. It has limited applications in cervical lymph nodes and systemic assessment. Besides, there are a series of TI-RADS classifications currently used in China. The same patient gets different TI-RADS classifications by different ultrasound doctors in different hospitals, which brings great confusion to patients [10–12]. At present, the vast majority of new thyroid cancers are very low malignant papillary thyroid microcarcinoma (PTMC), which is recommended for "active monitoring" without immediate surgery [13,14]. This study evaluated the T/N staging of DTC by ultrasound-based AJCC TNM criteria, and tried to evaluate the TNM staging of thyroid nodules in combination with neck chest enhanced CT in the future. As a supplement to TI-RADS classification standard, it is more conducive for clinicians to comprehensively evaluate the clinical risk of thyroid nodules and select appropriate treatment methods, to reduce the operation rate of DTC.

In this study, the criteria for determining T-stage by ultrasound were lesion size and whether the thyroid capsule invaded. The sensitivity and specificity of the ultrasonic T stage in assessing T1, T2 and T4 tumors were good, which is consistently higher than 65%. The PPV values were satisfying, higher than 50% in all these groups. These data suggest that was ultrasound T-stage could provide relatively reliable diagnostic information. Although the ultrasonic T-stage (T ≤ 2 stage) was evaluated mainly according to the nodule size, it still showed some discrepancy with the pathological T-stage. The reason may be that the contraction of pathological specimens will change the lesion size. One previous study showed that pathological samples are reduced by approximately 10% compared to ultrasonic measurement data [15], resulting in a reduction of the pathological T stage compared to the ultrasonic T stage. At the same time, different nodule shapes lead to differential measurement errors in different sections.

However, the sensitivity in T3b is low: 29.5%, 95%CI: 16.1%-43.0%. For ultrasonic T3a and T3b, the PPV are only 34.3% (12/35) and 20.3% (13/64) respectively. The reason for the low PPV of ultrasound T3 stage may be that the ultrasonic evaluation of thyroid capsule and capsule invasion has not established the diagnostic criteria and predictive indicators [16]. There is a large error in the ultrasonic evaluation of nodular thyroid capsule invasion and growth. Secondly, it is not clear whether the thyroid gland really has a capsule [17]. The pathologist has subjective judgment on the growth of capsule invasion, which might lead to the ultrasonic manifestation of capsule invasion. In 163 cases, only nearly half of 81 cases showed pathological invasion of the capsule. Thirdly, these low rates might be associated with the limited capability of ultrasound in detecting larger benign thyroid nodule, since over a half (19/35) of the ultrasonic T3a were actually pathological T1/2 (Table 4).

T4 stage is defined as a moderately progressive disease, which is obvious and typical in ultrasound imaging and pathology. Therefore, ultrasonic stage and high sensitivity, specificity and PPV in T4 tumors. Considering the highly reliable data, ultrasonic T staging is very suitable for clinical evaluation of thyroid micro nodules and typical capsule invasive growth nodules.

In terms of N-stage, the sensitivity, specificity and PPV for N0 tumors were 87.7% (95%CI: 85.1%-90.3%) 41.0% (95%CI: 37.0%-45.0%) and 61.0% (95%CI: 57.8%-64.2%) respectively. However, the consistencies between ultrasonic N stages and pathological N stages were poor (kappa value<0.3 in all N stages). Besides, the sensitivity, specificity and PPV were also significantly dropped. For N1a and N1b tumors, the PPV were 48.2% (95%CI: 38.8%-57.6%) and 24.3% (95%CI: 17.2%-31.4%) respectively. The first reason is that the central cervical region is difficult to be examined by ultrasound due to its deep location and blocked by the thyroid gland. Secondly, the sonogram of early cervical lymph node metastasis is not typical. It is difficult to distinguish by ultrasound. However, it can also truly reflect the lymph node situation in the neck region of more than half of thyroid nodules, especially for typical cases with ultrasonic signs of cervical lymph node metastasis [18]. A multicenter study of 4014 cases of thyroid cancer showed that, preoperative ultrasound showed poor sensitivity in the diagnosis of lymph node metastasis in the central region of the neck, and good effect in the diagnosis of lymph node metastasis in the lateral region of the neck [19]. At the same time, the transfer rates of incised lymph nodes in the central region and lateral region of the neck were 36.2% and 46.6% respectively. The rate of cervical lymph node metastasis was 48.7% (588 / 1206). Previous studies indicated that cervical lymph node metastasis of thyroid cancer has little impact on the overall survival rate [20,21]. Although the sensitivity, specificity and PPV were not satisfying, ultrasonic N staging had high specificity (>90%) and relatively high diagnostic accuracy (>70%) in N1 tumors, indicating that preoperative ultrasound evaluation of clinical N-stage has a certain clinical value for DTC risk assessment.

In terms of M-stage, the most common metastatic sites of thyroid cancer are lung and bone [22,23]. Therefore, the advantages of ultrasound cannot be brought into play. This is the reason why this study did not include M-stage assessment.

Based on our retrospective analysis, we infer that ultrasonic evaluation of DTC T/N staging has its advantages over simple TI-RADS classification. Firstly, if malignant thyroid nodules are identified as T1/N0 (equivalent to clinical low-risk thyroid nodules), the best management strategy is "active surveillance". This strategy might help avoid puncture biopsy or surgery recommendation for the TI-RADS 3, 4A, 4B, 4C and 5 cases. Secondly, if the malignant thyroid nodules are evaluated as T3N_0_ or T4N0 nodules (equivalent to clinically medium-risk thyroid nodules), the therapeutic strategy can be active monitoring treatment, puncture biopsy or surgical treatment according to the communication between doctor and patients. Finally, if the malignant thyroid nodule is evaluated as T_x_N_1_ nodule (equivalent to clinical high-risk thyroid nodule), the best therapeutic strategy is puncture biopsy or surgical treatment.

In conclusion, neck ultrasonography has its own advantages in assessing T1, T2 and T4 DTC. Although the sensitivity, specificity and PPV were not satisfying, ultrasonic N staging had high specificity and relatively high diagnostic accuracy in N1 tumors. Therefore, ultrasonic T/N staging might be considered for assessing malignant thyroid nodules, especially for thyroid micro nodules.

## Supporting information

S1 Table Clinical parameters of DTC cases included in this study. (XLSX)
